# Measuring Biomass and Carbon Stock in Resprouting Woody Plants

**DOI:** 10.1371/journal.pone.0118388

**Published:** 2015-02-26

**Authors:** Radim Matula, Lenka Damborská, Monika Nečasová, Milan Geršl, Martin Šrámek

**Affiliations:** 1 Department of Forest Botany, Dendrology and Geobiocoenology, Faculty of Forestry and Wood Technology, Mendel University in Brno, Brno, Czech Republic; 2 Department of Agriculture, Food and Environmental Engineering, Faculty of Agronomy, Mendel University in Brno, Brno, Czech Republic; Bangor University, UNITED KINGDOM

## Abstract

Resprouting multi-stemmed woody plants form an important component of the woody vegetation in many ecosystems, but a clear methodology for reliable measurement of their size and quick, non-destructive estimation of their woody biomass and carbon stock is lacking. Our goal was to find a minimum number of sprouts, i.e., the most easily obtainable, and sprout parameters that should be measured for accurate sprout biomass and carbon stock estimates. Using data for 5 common temperate woody species, we modelled carbon stock and sprout biomass as a function of an increasing number of sprouts in an interaction with different sprout parameters. The mean basal diameter of only two to five of the thickest sprouts and the basal diameter and DBH of the thickest sprouts per stump proved to be accurate estimators for the total sprout biomass of the individual resprouters and the populations of resprouters, respectively. Carbon stock estimates were strongly correlated with biomass estimates, but relative carbon content varied among species. Our study demonstrated that the size of the resprouters can be easily measured, and their biomass and carbon stock estimated; therefore, resprouters can be simply incorporated into studies of woody vegetation.

## Introduction

Resprouting trees and shrubs are often an important component of woody plant vegetation from the temperate zone to the tropics [1, 2–6]. Resprouting woody plants are also used in expanding, short-rotation coppices grown for rapid woody biomass production as a source of renewable energy [7–10]. The amount of woody biomass is a key variable for many purposes, such as assessing the woody plant productivity, carbon storage or economic value of wood in forests and plantations, but it is not directly measurable; for this reason, a number of methods for indirect woody biomass estimation have been published [11, 12–15]. However, the vast majority of these methods have been developed for “classic” one- or a few-stemmed trees, whereas in spite of the dominance of multi-stemmed resprouters in many ecosystems, the basic methodological steps for accurate sprout biomass and carbon stock estimation, such as which parameters and how many sprouts to measure, are lacking at both the individual and stand levels. This lack of unified methodology leads to differences in measured sprout parameters among studies, which causes their results to often not be comparable and may also result in a bias in the estimated woody biomass and the biomass-related variables.

The lack of a common and reliable parameter representing the size of the whole individual for resprouting woody plants, such as diameter at breast height (DBH) or height in one-stem trees, arises from the structural complexity of multi-sprout trees, which usually consist of many stems (sprouts), especially for young trees. For example, Matula et al. [16] found a resprouting *Tilia cordata* tree one year of age with more than 400 sprouts and many other trees with more than 100 sprouts; Mosseler et al. [17] reported > 40 sprouts in coppiced willows. Measuring each individual sprout for such resprouting trees to estimate their total woody biomass is therefore very time-consuming and ineffective, especially compared with one-stem tree measurements. In addition, every study that involves woody plant measurements sets a minimum size, usually a minimum stem diameter or height, for these plants to be recorded. However, the total woody biomass contained in one stem of a one-stemmed tree is divided into several smaller stems in a resprouting tree, which implies that the stems of the resprouting tree might be smaller than the minimum measurable size and thus would not be measured, whereas a one-stem tree with the same biomass would be. Moreover, even if some of the sprouts were greater than minimum measurable size, many others of the same individual would not be, and thus, only a portion of the entire biomass of the resprouting trees would be captured. The proportion of uncaptured sprouts, and thus of uncaptured biomass, would most likely increase with the increasing sprout biomass of the resprouting trees because number of sprouts per individual is positively correlated with the total above-ground biomass of a resprouter [16–18]. For all these reasons, the biomass and the related carbon stock of the resprouters is likely to often be underestimated, which also implies that the above-ground biomass estimates of all woody plants or the estimated carbon balances might be significantly biased in a number of studies, especially in ecosystems dominated by woody vegetation.

Most studies that have dealt with sprout biomass estimates have primarily calculated allometric equations for the individual sprouts [10, 19–21], and the total sprout biomass has been counted as the sum of the biomasses of the individual sprouts, which does not solve the problem of inefficient measurement of the whole resprouting individual. However, a recent study by Mosseler et al. [17] found that biomass estimates based on sprout length and the diameter of the 3 largest sprouts were more precise than estimates based on the 20 largest sprouts, which indicates that the biomass of all sprouts can be estimated using the parameters of only a few of the largest sprouts. However, which parameters are better biomass predictors and the effect of the number of sprouts measured on the accuracy of biomass estimation remain unclear.

The carbon stock of woody plants is often calculated simply as half of the woody biomass [12, 22], but some studies have shown that this approach is over-simplified and that there is significant intra- and inter-specific variation in the ratio between tree biomass and carbon content, ranging from 44.4% to 55.7% depending upon tree species and biomass tissues [23–25]. For sprouts, the information on such variation is lacking, although it must be considered for reliable carbon stock estimates in resprouting trees.

In this study, our objective was to resolve all of the above-mentioned issues. Using data for the different sprout parameters, sprout biomass and the carbon stock of 5 common temperate woody species, we tested how the accuracy of sprout biomass and carbon stock estimates changes with the use of different sprout parameters for the estimation and with varying numbers of sprouts (per individual) on which the parameters are measured. The goal was to find both species-specific and general minimum numbers of sprouts, i.e., the most easily obtainable and the most appropriate parameter to measure to obtain accurate individual and stand-level sprout biomass estimates. To determine how much accuracy would be sacrificed by using only some instead of all of the sprouts for biomass estimation, we compared the biomass estimates based on a few representative sprouts with the estimates based on the data for all sprouts, i.e., with the most accurate estimates expected but requiring the maximum possible effort in the field. In addition, to test all of the relationships, we created and provide in the supplementary materials several species-specific and general allometric equations, which can be used for direct calculations of the biomass and carbon content in sprouts.

## Materials and Methods

The Training Forest Enterprise Křtiny of Mendel University in Brno (TFE) as the land owner of the study area granted permission for the access to the plots and for field data collection. TFE (slp@slpkrtiny.cz) also should be contacted for permission for any future research activities in the area. The species involved in our study were neither endangered nor protected.

This study was performed in two study plots, Hády and Soběšice, located in TFE, in the southeastern Czech Republic (49°13'30''N, 16°40'55''E and 49°14'43"N, 16°35'59"E, respectively). Each plot has an area of 4 ha. The elevation of the study areas is 401 m a.s.l. in Hády and 355 m a.s.l. in Soběšice. The bedrock is chalk in Hády and granodiorite in Soběšice, and the soils are brown forest soils in Hády and cambisols in Soběšice. The average annual rainfall is 510 mm, and the average annual air temperature is 8.4°C in both plots. The average temperature in July (the warmest month) is 18.4°C, and the average temperature in January (the coldest month) is −2.1°C, based on data from 1960–2010 from the Brno weather station (the nearest weather station for both plots).

In 2009, the original old growth forests in both plots were harvested, but some residual trees were left standing in densities ranging from 0 to 200 trees per hectare. The residual trees averaged 21.1 m in height and 41.5 cm in diameter at 1.3 m (DBH). Both plots were fenced because there was significant game pressure in the area, and no management has been applied since the harvest.

In winter 2012/2013, four years after the harvest, we selected 120 resprouting trees (stumps) of four common tree species (30 per species) and 30 resprouting individuals of a shrub species. The tree species were sessile oak (*Quercus petraea* (Matt.) Liebl.), small-leaved lime (*Tilia cordata* Mill.), European hornbeam (*Carpinus betulus* L.) and field maple (*Acer campestre* L.), and the shrub species was common hazel (*Corylus avellana* L.). All species were deciduous. Because all standing trees and resprouting stumps are fully mapped in the two plots, and their sprouts/stems are regularly measured, we were able to select resprouters that were scattered regularly across the 8 ha area of the two study plots (15 per plot and species) and in the desired range, from individuals with only a few small sprouts to individuals with a large number of large sprouts for each species. The resprouters were selected in areas with and without standing residual trees to create variability in the light conditions, but the light variability was not included in the analyses because we wanted our allometric equations for biomass calculation to be as general as possible. The number of sprouts per individual ranged from 4 to 61; the ranges of different sprout size variables of measured woody plants are shown in Table 1. For each selected individual, each sprout of the stump was cut, and its length (L_indiv_), diameter at 5 cm above its base (BD_indiv_) and diameter at 1.3 m (DBH_indiv_) were measured. Most sprouts grew directly from the stump or the stump collar. The diameters were measured with calibrated digital callipers ABS SOMET with a precision of ± 0.1 mm. All of the removed sprouts were cut up into small pieces and dried at 80°C until reaching constant weight and then weighed. Three sprouts from each stump—one large, one medium and one small (selected by observation)—were stored and weighed separately to create the allometric equations for the individual sprouts. In addition, we measured the sprout carbon content of each measured individual by grinding up the entire medium sprout into powder and then measuring the total organic carbon bound in an organic compound (hereafter referred to as carbon content) using high-temperature combustion. The samples were combusted at 1200°C in a pure oxygen atmosphere. In this method, all carbon is converted into carbon dioxide, which is then analysed with an Infrared Detector. For the calibration procedure, we used calcium carbonate and graphite. The analysis was performed using an Analytik Jena, Multi N/C 2100 and HT 1300 device.

**Table 1:** Ranges of measured values of basal diameter of individual sprouts (BD), diameter at breast height of individual sprouts (DBH), length of individual sprouts (L), basal area of all sprouts per stump (BA) calculated from BD, and biomass of all sprouts per stump (Biomass).

Species	Size ranges				
	BD (mm)	DBH (mm)	L (cm)	BA (cm^2^)	Biomass (kg)
Pooled	2.5–84.8	1.1–55.4	29–673	4.1–189.9	0.16–16.28
*Acer campestre*	3.2–48.5	1.4–30.1	42–450	6.2–122.3	0.47–13.94
*Carpinus betulus*	3.7–42.5	1.5–27.6	54–450	6.9–73.0	0.30–6.54
*Corylus avellana*	2.5–84.8	1.1–55.4	29–673	4.1–189.9	0.16–16.28
*Quercus petraea*	4.7–48.5	2.3–29.8	38–391	8.5–84.8	0.47–12.38
*Tilia cordata*	5.2–39.6	1.3–27.7	66–365	8.7–132.5	0.30–6.55

### Data Analysis

Because the individuals measured were mostly cut stumps that resprouted, we refer to them hereafter as stumps. For each stump, the sprouts were sorted from the largest to smallest for a given sprout parameter (L, DBH and BD). Then, for each parameter, we recorded the value of the largest sprout, then the average value of the two largest sprouts, then the average value of the three largest sprouts, and so forth, up to the average value of the ten largest sprouts. The average values for more than 10 sprouts were not used because many stumps had approximately 10 sprouts, and thus all sprouts would have been measured, which would not be an improvement of the measurement method. In stumps with less than 10 sprouts, we used the average of all sprouts as the average parameter value for ≥ the number of sprouts of such stumps, e.g., for a stump with 8 sprouts we used the average of all 8 sprouts as the average value of 8, 9, and 10 sprouts.

The averaged parameter values (L_avg_, DBH_avg_ and BD_avg_) were then used as independent variables in non-linear models (i.e., allometric equations) that we created to predict the total sprout biomass per stump (Biomass_avg_) and the total carbon content per stump (C_avg_). Using the sum or any other parameter “transformation” instead of the average would have the same accuracy, but in our opinion, the average gives the most intuitive values. A visual evaluation of the plotted data suggested that they followed either exponential or power law exponential functional forms, which therefore were fitted to the data and compared. The formulas used for the two models were:

- a) Exponential: *y = ae* ^*bx*^
- b) Power law: *y = ax* ^*b*^

where y is the response variable (Biomass_avg_ or C_avg_), x is the average value of the given parameter (BD_avg_, DBH_avg_ and L_avg_) for the given number of largest sprouts (1–10) within a stump, and a and b are the model coefficients. For each model we calculated Root Mean Squared Error (RMSE), second-order Akaike Information Criterion (AIC_c_) [26] and likelihood-ratio based pseudo-R^2^ [27] with Nagelkerke’s adjustment (pseudo-R^2^_adj_) [28]. We calculated differences between AIC_c_ values (ΔAIC_c_) for exponential and power law models and selected those with lower AIC_c_ values. The other possible functional forms in Paine et al. [29] were also tested, but they had much worse fits to the data, with ΔAIC_c_ ≥ 10.11, compared with the exponential or power law models and were not considered further in this study. The models were created and tested both for the individual species (species-specific models) and for the pooled data (general models). Because there was apparent heteroskedasticity in the majority of the data, we used generalized non-linear models (GNLMs). Because exponential and power law models may be linearized through log-transformation of the response variable or log-transformation of both the response and explanatory variables, respectively [15], we also tested this technique to fit the models because fitting linear models is usually easier than direct fitting of nonlinear models. However, the linearized models had clearly worse fits to our data compared with the GNLMs, i.e., they had higher RMSE values in most of the models. Therefore, we used the coefficients from these linearized models only as starting values for fitting the GNLMs. To test for differences in the model coefficients among species and sites, we created models with sites and species in the interaction with a given sprout parameter and, using analysis of variance (ANOVA), compared these models with models in which species and sites were pooled. The coefficients for sites and species of such models did not differ when the models in which they appeared in the interactions did not differ from the models with pooled data (P ≥ 0.05). To calculate sprout biomass estimates for several stumps together, such as for plot-, stand- or population-level estimates (hereafter referred to as stand-level biomass estimates—Biomass_stand_), we simply summed Biomass_avg_ for the species and for the pooled data and compared those sums with the sums of the weighted sprout biomasses per stump (Biomass_weighted_).

To calculate the total sprout biomass per stump based on the measurements of all the sprouts, we created species-specific and general GNLMs for individual sprout biomass estimates (Biomass_indiv)_ using the biomass of the individual sprouts as the response variable and the measured parameters as the explanatory variables. The model selection and evaluation were performed exactly as for the averaged sprout parameter (see paragraph above). The estimated total biomass per stump (Biomass_sumindiv_) per species and pooled data were obtained by summing the Biomass_indiv_ values, which were calculated using the species-specific and general individual-sprout biomass models with the highest pseudo-R^2^_adj_ values. Using the total Biomass_sumindiv_ and Biomass_weighted_, RMSE values were computed for each species and for the pooled data. Because C was measured as a proportion of the biomass for each stump, its estimates were not calculated with all sprouts; the difference between such models would have been the same as the difference between the total Biomass_sumindiv_ and Biomass_avg_ models and thus would not provide any additional information. The models with DBH as a biomass predictor were also not calculated with all sprouts because many sprouts were not 1.3 m in length, and thus the total biomass would be underestimated.

All analyses were performed in R [30] using the “nlme” package [31]. Model selection, fitting and evaluation followed the recommended procedures and R script from Paine et al. [29]. Model coefficients and equations for biomass and carbon stock calculation are provided in S2 Table.

## Results

### Estimates based on the averaged parameters of the largest sprouts

**Fig 1:**
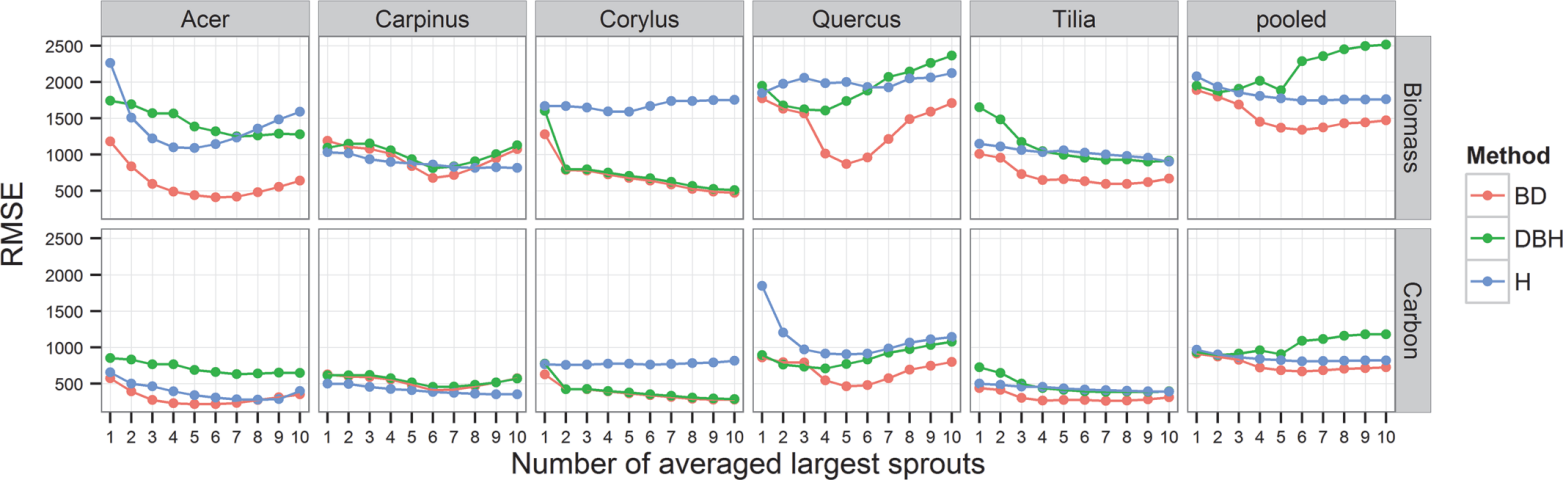
Relationship between RMSE values of the generalized exponential models for sprout biomass and the carbon stock (per stump) estimations and the number of largest sprouts averaged for the given parameter (BD_avg_, DBH_avg_ or L_avg_) used as an estimator in the models.

The Biomass_avg_ and C_avg_ were better estimated by the exponential models than by the power law models in all species, predictors and explanatory variables (ΔAIC_c_ ≥ 4.45). All three sprout parameters of the largest sprouts were significantly correlated with the Biomass_weighted_ (P < 0.05), but the accuracy of the Biomass_avg_ and C_avg_ estimates based on them varied among the species and with the number of averaged largest sprouts (Fig. 1, S1 Table). There was no significant difference between the model coefficients for the two sites (P ≥ 0.224) or between *Acer* and *Carpinus* (P > 0.05). The differences between the other species depended on the parameter used. *Quercus* was significantly different from the other species in the models with L_avg_ (P = 0.039) and DBH_avg_ (P < 0.001), *Corylus* in the models with BD_avg_ (P < 0.001) and DBH_avg_ (P < 0.001) and *Tilia* in the models with BD_avg_ (P < 0.001). For *Carpinus*, BD_avg_, DBH_avg_ and L_avg_ performed similarly as predictors, as did DBH_avg_ and BD_avg_ for *Corylus*; however, in all other species, BD_avg_ gave the best estimates (Fig. 1). In comparison with DBH_avg_, L_avg_ was a better predictor for *Acer* but was clearly worse for *Corylus*, *Quercus*, *Tilia* and the pooled data (Fig. 1).

**Fig 2:**
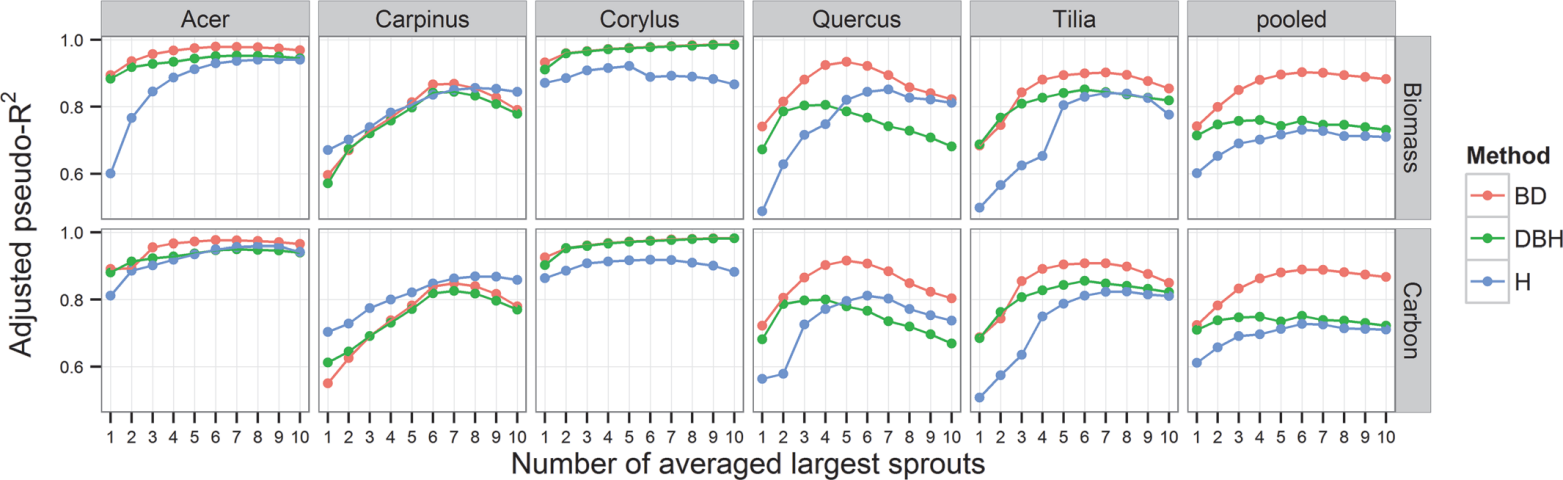
Relationship between the pseudo-R^2^_adj_ values of the generalized exponential models for sprout biomass and the carbon stock (per stump) estimations and the number of largest sprouts averaged for the given parameter (BD_avg_, DBH_avg_ or L_avg_) used as an estimator in the models.

**Fig 3:**
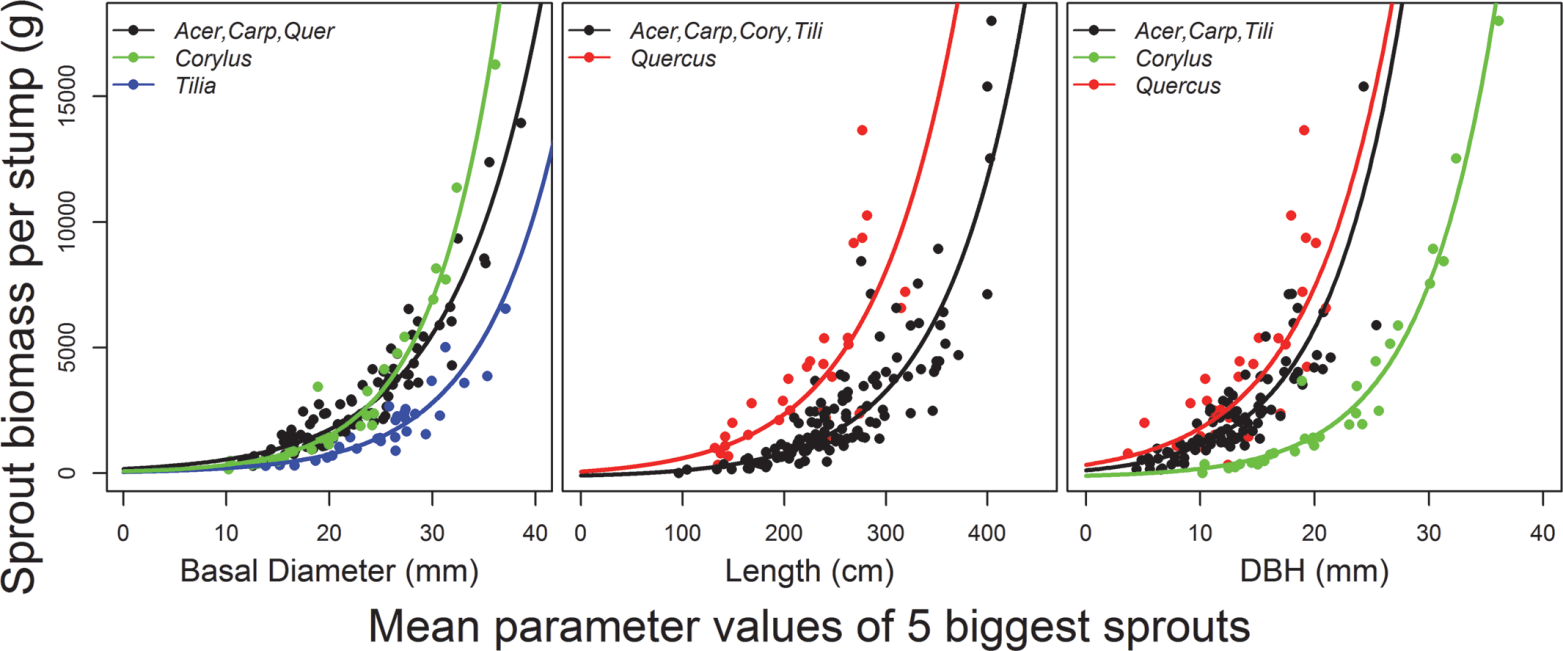
Prediction of sprout biomass per stump based on averaged BDs and DBHs of 5 thickest sprouts per stump and based on averaged L of 5 longest sprouts. Exponential model was used for ploting lines. Species without significant difference in slope (P > 0.05) are shown together. Parameter coefficients and goodness of fit statistics for each model and species are shown in S1 Table.

The accuracy of the estimation using BD_avg_ greatly increased with each sprout added to the BD_avg_ up to the two averaged thickest sprouts for *Corylus*, three for *Acer* and *Tilia*, four for *Quercus* and the pooled data, and up to five for *Carpinus* (Fig. 1). Neither RMSE nor AIC_c_ values greatly decreased nor pseudo-R^2^_adj_ values increased after five or six averaged sprouts in any of the species in the models with BD_avg_ (Figs. 1, 2 and S1 Fig.); therefore, 5 was the minimum number of thickest sprouts to approach the maximum or near-maximum accuracy for all species, and the exponential model with five sprouts generally predicted Biomass_avg_ well (Fig. 3). For the other parameters, there was no clear minimum number of largest sprouts because of the large variation in the maximum pseudo-R^2^_adj_ and RMSE values among the species (Figs. 1 and 2), but the average of 5 sprouts also predicted Biomass_avg_ well (Fig. 3). With the exception of *Carpinus*, the accuracy of the estimates decreased as the number of averaged largest sprouts increased toward ten. This decrease was most pronounced in *Quercus*, for which the accuracy of the estimation decreased with ≥ 6 averaged largest sprouts (Fig. 1).

**Fig 4:**
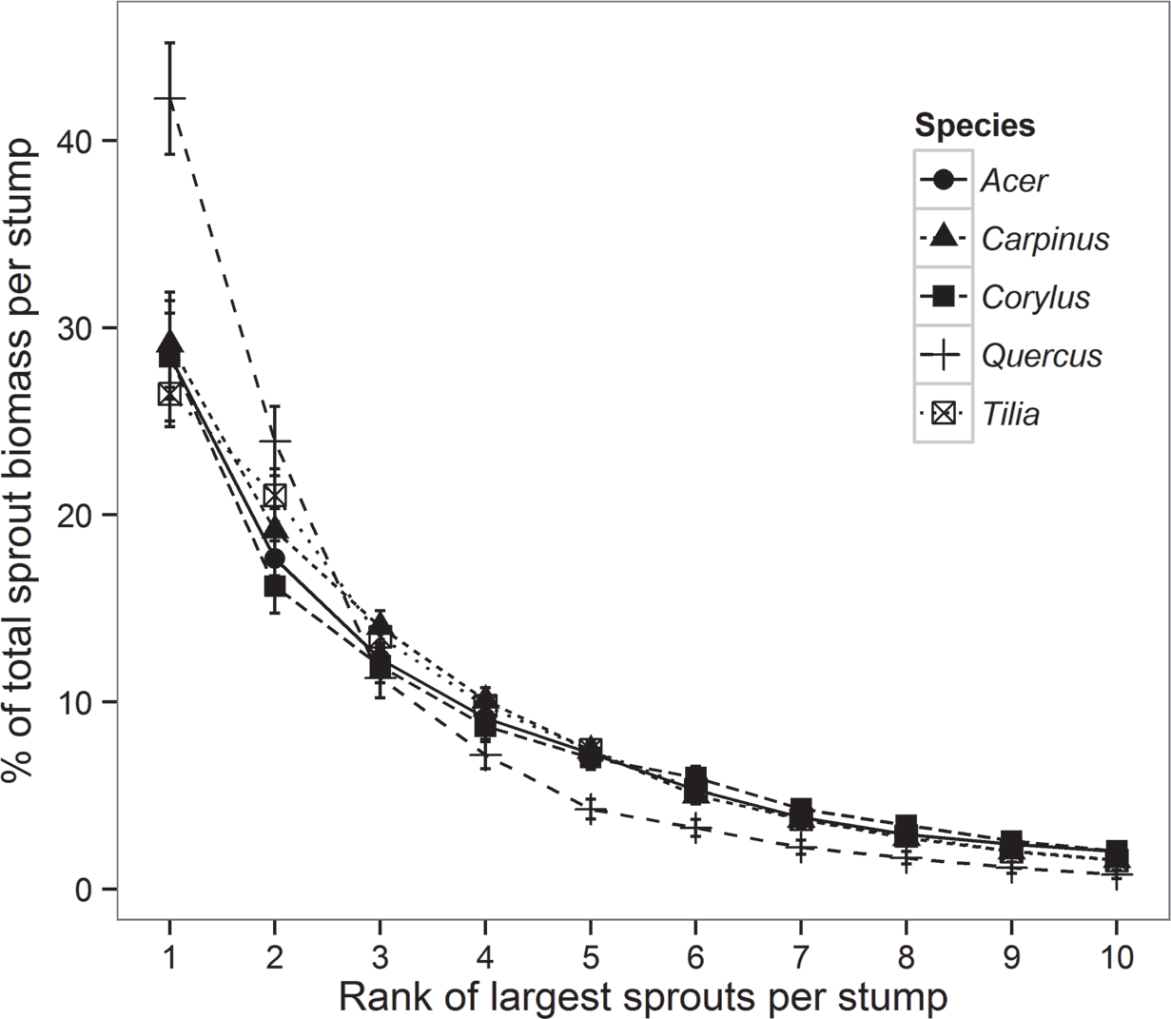
Mean relative contribution of the individual largest sprouts per stump to the total sprout biomass ranked according to their size rank per the stump, e.g., 1 indicates the largest sprout, 2 the second largest sprout, etc.

The contribution of an individual sprout Biomass_indiv_ to the total sprout biomass per stump decreased exponentially with decreasing size rank of the sprout among all sprouts on the stump; i.e., the largest sprout had the greatest biomass, the second largest sprout had the second greatest biomass, etc. (Fig. 4). For this reason, the majority of the total biomass was located in a few of the largest sprouts (S2 Fig.); for example, more than half of the total sprout biomass per stump was located in only the 3 largest sprouts, and ≥ 80% of the total sprout biomass was located in the six largest sprouts for all species (S2 Fig.).

**Fig 5:**
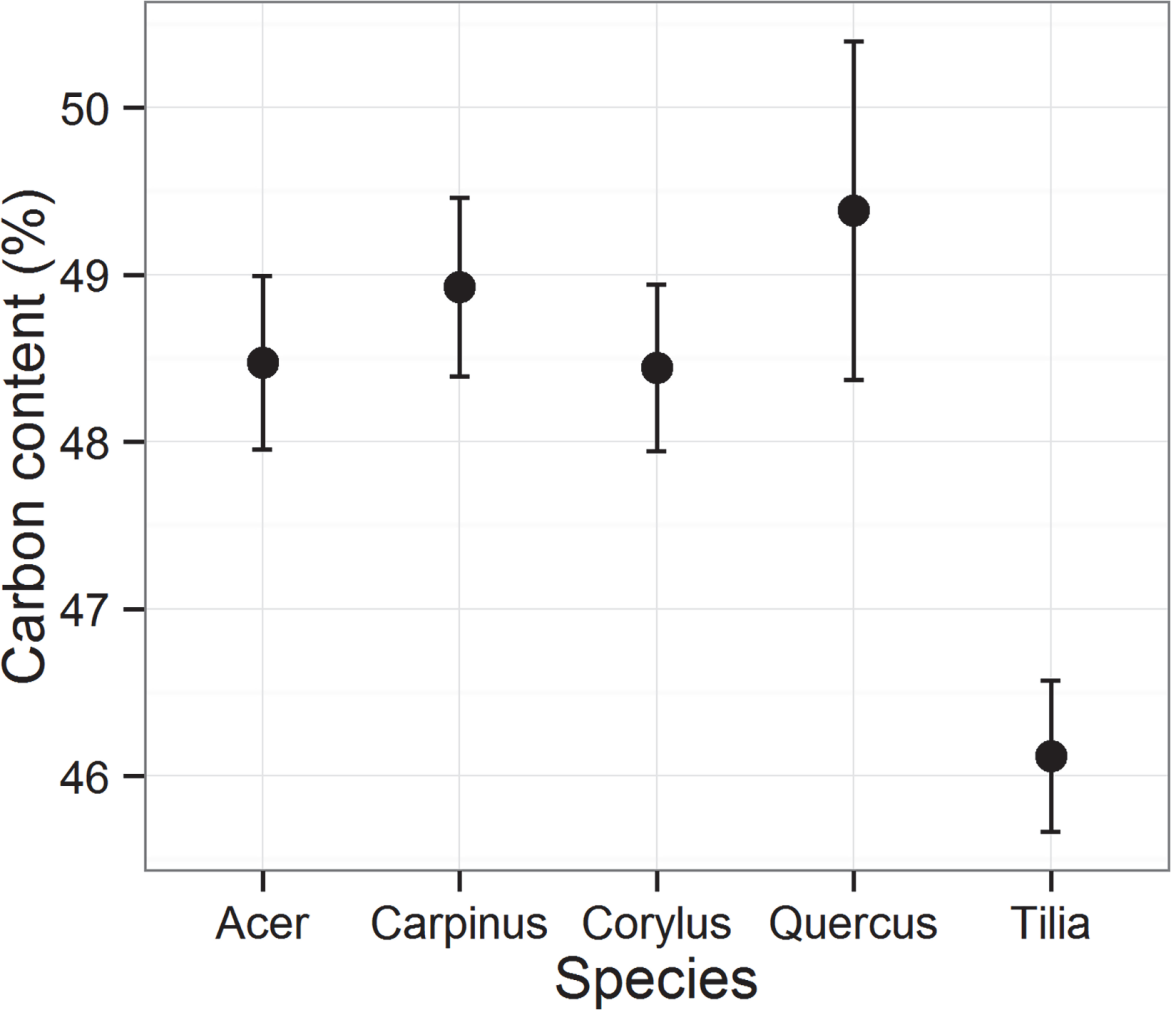
Relative carbon content of dried woody biomass of sprouts.

RMSEs and pseudo-R^2^_adj_ values of models for C_avg_ were strongly correlated with RMSE and pseudo-R^2^_adj_ values of Biomass_avg_ models (Figs. 1and 2), but there was significant variation in the relative content of C with species (Fig. 5). The relative C content ranged from 48.4 to 49.4% of the woody biomass in *Acer*, *Carpinus*, *Corylus* and *Quercus* but it was significantly less in *Tilia* than in the other species (46.2%; P < 0.001; Fig. 5). The other species did not significantly differ from each other (P > 0.05).

### Estimates of sprout biomass at the stand level

**Fig 6:**
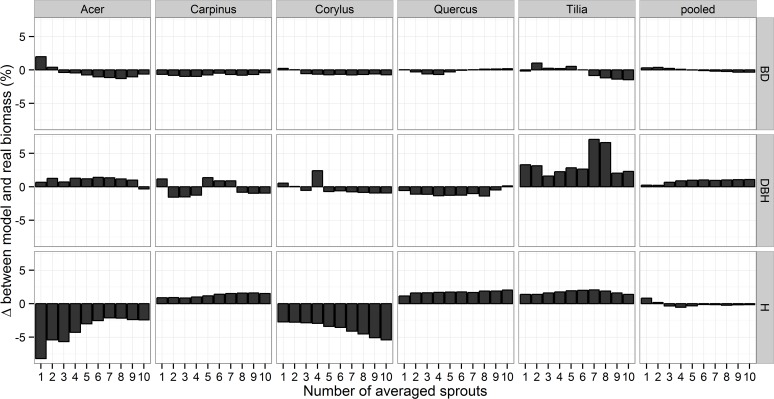
Relative differences between the total model biomass (Biomass_stand_) and the total weighted biomass (shown as grey bars) of all sprouts per species and of all 5 species together (pooled) in relation to the number of averaged largest sprouts and a parameter used for estimation. For the averaged basal diameter (BD) and the diameter at breast height (DBH), the 1–10 thickest sprouts were used, and for the average length (L), the 1–10 longest sprouts within a stump were used.

The summed values of the predicted Biomass_stand_ for each species generally differed by ≤ 2% from the real total biomass per species (Fig. 6). Such accurate estimates were obtained in the majority of species using parameters of only the largest sprout per stump. The only exceptions to the ≤ 2% accuracy, i.e., the worst estimates, were those based on the height of the 1–5 tallest sprouts in *Acer*, 5–10 tallest sprouts in *Corylus* and the averaged DBHs of 1, 8 and 9 sprouts per stump in *Tilia*, but even the worst estimate differed from the real value by only 8.1% (Fig. 6). The predicted total summed biomass for the pooled data was even more accurate than the species-specific estimates (Fig. 6) because the worst estimate differed only by 1.08% from the real biomass, an error of 4.5 kg of the total 414.8 kg.

### Estimates based on all sprouts

Biomass_indiv_ was better fitted by the power law model for all parameters and species (ΔAIC_c_ ≥ 5.52; S3 Fig.). BD_indiv_ was the best estimator of Biomass_indiv_, and models that included it had high pseudo-R^2^_adj_ values (> 0.90) for all species, although the pseudo-R^2^_adj_ for the pooled data was slightly lower (S2 Table). Compared with the BD_indiv_, the models with DBH_indiv_ produced worse estimates, especially for the pooled data and *Acer*, but L_indiv_ was the worst predictor (S2 Table). The regression coefficients of *Carpinus* and *Acer* did not significantly differ (P = 0.124).

BD_indiv_ and L_indiv_ estimated Biomass_sumindiv_ well (S3 Table). BD_indiv_ was a better predictor of Biomass_sumindiv_ than was L_indiv_ for *Acer*, *Corylus* and *Tilia*, but BD_indiv_ was a worse predictor for *Quercus;* for the pooled data and *Carpinus*, both predictors performed similarly (S3 Table). In comparison with the estimates obtained using the averaged parameters of the 5 largest sprouts, the Biomass_sumindiv_ estimates were more accurate for *Carpinus*, *Tilia* and *Corylus*, and less accurate for the pooled data, *Quercus* and *Acer* (S3 Table).

## Discussion

Our study showed that in spite of the structural complexity of multi-sprout woody plants, their biomass and carbon stock can be reliably estimated using the parameters of only one or a few largest sprouts, depending on whether the estimation is for an individual or for a stand. We demonstrated that, to achieve the best or close to the best possible sprout biomass estimates of an individual resprouter, the basal diameters of the 2 to 5 largest sprouts need to be measured; however, at the stand level, only the parameters of the largest sprouts are needed for nearly perfect estimates with almost no error. Surprisingly, our results revealed that using the parameters of more than five or even all of the sprouts of a stump may result in no significant increase in the accuracy of the biomass estimates, and in some species, such as *Quercus* and *Carpinus*, it may even lead to less precise estimates than measuring only five or fewer of the largest sprouts.

In the 5 species in our study, the mean parameter values of only a few of the largest sprouts per resprouter correlate better with the biomass of all sprouts than the mean parameter values of all or many sprouts, which corroborates Mosseler et al. [17], who reported more accurate estimation of sprout biomass with 3 compared with 20 sprouts in *Salix*. Because such a correlation between the average size of a few (≤ 5) of the largest sprouts and the biomass of all sprouts has been found in several species of resprouting shrubs (*Salix*, *Corylus*) and trees (*Acer*, *Carpinus*, *Quercus*, *Tilia*), the correlation is likely to hold for resprouting woody plants in general and thus can also be conveniently used for biomass estimation in other species.

The good prediction of biomass and carbon stock using 5 or fewer of the largest sprouts is most likely linked to the size distribution of the sprouts within a stump which is determined by intra-specific (within stump) sprout competition, causing a few sprouts to gain dominance and suppress the remaining sprouts [18, 32]. The 5th largest sprout seems to be a “threshold” in this sprout size distribution; for sprouts within a stump ranked by their size from smallest to largest, the proportion of each sprout in the total sprout biomass increased only slightly with increasing size rank up to the rank of the 5th largest sprout, whereas from the 5th largest to the largest sprouts, there was a rapid increase in the contribution to the sprout biomass with each size rank. Therefore, most of the sprout biomass is contained in the 5 largest sprouts, which thus best offset the biomass of the entire resprouting individual. By contrast, the remaining smaller sprouts seem to not be representative of the total sprout biomass, and thus, when they are averaged with the largest sprouts for biomass prediction, they may significantly bias the resulting biomass estimates. In addition, because the 5 largest sprouts are significantly bigger than the remaining sprouts they can be easily identified within a plant and measured.

However, there were inter-specific differences in the sprout size-rank representation of the sprout biomass. In *Acer* and *Corylus*, the estimates of sprout biomass per stump based on only the first or second largest sprouts were already highly accurate (pseudo-R^2^_adj_ ≥ 0.93), and using the parameters of the smaller sprouts for the estimation led to only similar or slightly better accuracy; in *Quercus*, *Tilia* and *Carpinus*, the parameters of 3 to 5 of the largest sprouts had to be used to obtain estimates with pseudo-R^2^_adj_ values > 0.80, and at some point, adding smaller sprouts to the estimation caused a decrease in pseudo-R^2^_adj_. Evidently, in *Quercus*, the few largest sprouts contained a greater proportion of the total sprout biomass than in the other species and thus were more representative of the total sprout biomass. Nevertheless, in the other species, the size rank distribution of the biomass within a stump did not differ and, therefore, cannot explain the differences among the species but we speculate that they are caused by variation in sprout architecture. When measuring sprouts in the field, we noticed that most sprouts in *Acer* and *Corylus* were straight, with a small, branched main stem growing upward, whereas in *Quercus*, *Tilia* and *Carpinus*, the sprouts often branched into multiple stems and/or many were tilted or even lying on the ground. Some of these irregular, tilted sprouts thus may be unrepresentative of the total stump biomass and may negatively affect the accuracy of the estimates but when their size is averaged with the others, this negative effect seems to be eliminated. Such information may be important for the inference of how many sprouts should be measured to obtain the desired level of accuracy for a biomass estimate of woody species not studied here. As our results indicate, if one wants to estimate the sprout biomass of woody species with “visually” regular sprouts with one clear stem, it is only necessary to obtain the parameters of the first or second largest sprouts, whereas for other species with non-regular sprouts, the measurements of 3 to 5 of the largest sprouts are needed.

Among the sprout parameters tested as biomass and carbon stock predictors, basal diameter proved to be the best, most universally reliable parameter. Although length performed as well as basal diameter in *Acer* and *Carpinus* as did DBH in *Carpinus* and *Corylus*, these two parameters provided mostly less accurate estimates in other species. Both the sprout basal diameter and the DBH have commonly been used as predictors in individual sprout biomass allometric equations [7, 10, 19, 20, 33, 34], but our results showed that basal diameter is clearly a better predictor than DBH and should be preferred if possible. The superiority of BD over DBH is likely because sprouts in the basal area are not branched, and thus, their BD represents the whole sprout; in contrast, from the basal area to breast height (1.3 m), the main stem often has several branches that are not generally measured, and thus, DBH is likely to underrepresent the biomass of the whole sprout. Similarly, sprout length might be a poor biomass predictor because sprouts may be broken or their tips killed by frost, fungi or herbivore infection, and thus, sprout length may not be representative of the whole sprout.

Our study also showed that sprout biomass and carbon stock at a stand level, i.e., for several resprouting trees together, can be very precisely estimated by summing the individual tree estimates calculated with the parameters of only the largest sprouts per stump. Compared with other methods for stand- or plot-level estimates [35], our method is the most precise to date and uses data that are easily obtainable in the field. In addition, the best estimates were obtained for the pooled data with the general equation, indicating that this method can be applied without identifying the species involved. This generality could be convenient, for example, in a tropical forest where resprouting is common [5] but it is impossible to create species-specific allometric equations due to high woody species diversity [11]. Evidently, the greater accuracy of the stand-level estimates compared with the individual stump estimates arose from the statistical methods used because the models for stump estimates were fitted using methods to minimize the sum of the residual errors. Therefore, if the model is correctly fitted, its sum of residual errors is zero or very nearly zero [36], and thus, most errors in the estimates of stump sprout biomass cancel out when the stump-level estimates are summed, resulting in the high accuracy of the stand-level estimates. In addition, as increasing numbers of stumps are summed, the errors are more likely to cancel out, and thus, the pooled data models in our study were more accurate (150 stumps in total) in comparison with the species-specific models (30 stumps per species).

As expected, the accuracy of the carbon stock estimates was closely correlated with the accuracy of the biomass estimates, which suggests that the sprout carbon stock can be calculated simply as a proportion of the woody biomass. However, the average relative carbon content was around 49% of the woody biomass in four of the five studied species whereas it was only 46.2% in *Tilia;* thus, if the carbon stock were simply calculated as half of the total woody biomass, as is commonly done [23, 24], carbon stock would be significantly overestimated. This stresses the importance of species-specific carbon content values for unbiased carbon stock estimates. Because related species with similar wood properties are likely to have comparable relative C contents [23] and our studied species represent some of the most common genera of broadleaved temperate species in the Northern Hemisphere, the relative sprout C contents reported in our study can be applied widely in temperate ecosystems.

Based on our results, we suggest that, for biomass estimates and for the representation of the size of individual resprouting woody species in multispecies forest, the basal diameter of 5 thickest sprouts of each individual should be measured. Measuring fewer sprouts would not lead to less accurate biomass estimates in some species, but it would in many others; thus, if the correlation between the largest sprouts and the total sprout biomass has not been tested for a given species, the 5 thickest sprouts “rule” should be followed to avoid the risk of greater error. For sprout biomass estimates at a stand level, we recommend measuring the basal diameter or DBH of the thickest sprout within each resprouting stump (or clump) in a plot and then calculating and summing the estimated sprout biomasses of the individual stumps. These methodological steps illustrate how easily the size of resprouters can be measured and their biomass and carbon stocks estimated and thus demonstrate that resprouters can be simply incorporated along with reseeders into studies of woody vegetation.

## Supporting Information

S1 Fig Relation between the AIC_c_ values of the generalized exponential models for sprout biomass and the carbon stock (per stump) estimations and the number of largest sprouts averaged for the given parameter (BD_avg_, DBH_avg_ or L_avg_) used as an estimator in the models. (TIF)

S2 Fig Mean relative contribution of summed largest sprouts per a stump to total sprout biomass ranked according to their size rank within stump, i.e. 1 stands for the largest sprout, 2 sum of two largest sprouts. (TIF)

S3 Fig Prediction of biomass of individual sprouts based on different sprout parameters. Power-law model was used for ploting lines. Species without significant difference in slope (P > 0.05) are shown together.
(TIF)

S1 Table Allometric equations for direct calculation of total sprout biomass and carbon stock per stump using different number of largest sprouts. (XLSX)

S2 Table Pseudo-R^2^_adj_ values of generalized Power law models for estimation of individual sprout biomasses based on basal diameter, DBH and sprout length. (XLSX)

S3 Table RMSE values for the models estimating the total biomass per stump by summing the estimated biomasses of the individual sprouts (Biomass_indiv_), calculated using the best individual-sprout models (RMSE-all). The RMSE values of the models estimating the biomass per stump using the average BD of the 5 thickest sprouts and the average L of the 5 longest sprouts are shown for comparison (RMSE-5).
(XLSX)
