# Supplementary figures and images for: Measuring Biomass and Carbon Stock in Resprouting Woody Plants
Source: PLoS One. 2015 Feb 26;10(2):e0118388. doi: 10.1371/journal.pone.0118388 (PMC4342014; doi:10.1371/journal.pone.0118388)

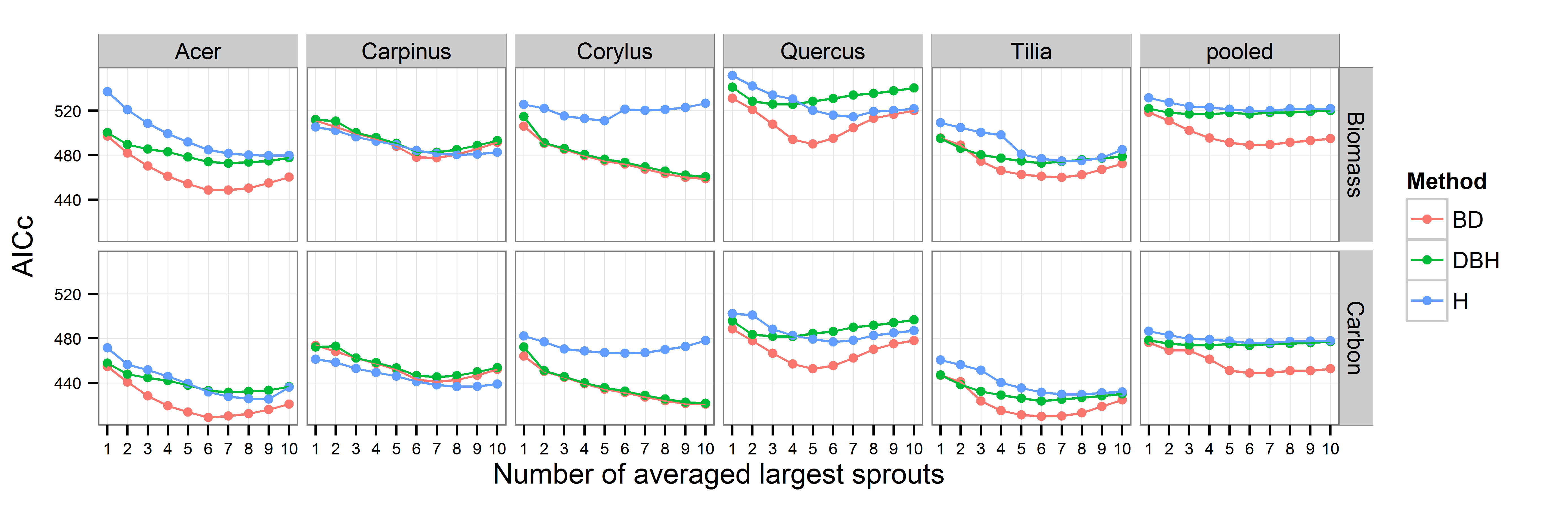

Supplement: S1 Fig — (TIF) [file pone.0118388.s001.tif]

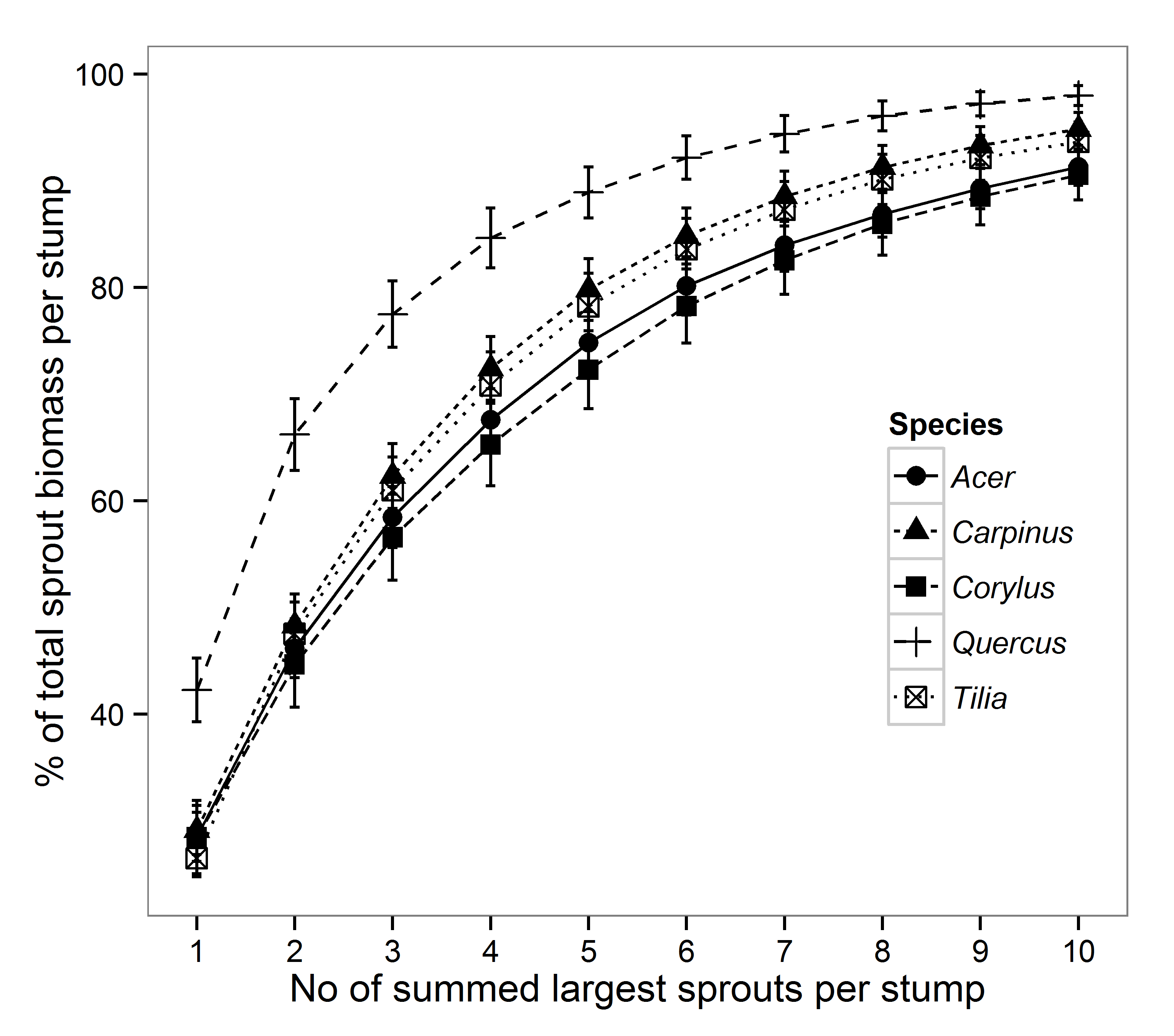

Supplement: S2 Fig — (TIF) [file pone.0118388.s002.tif]

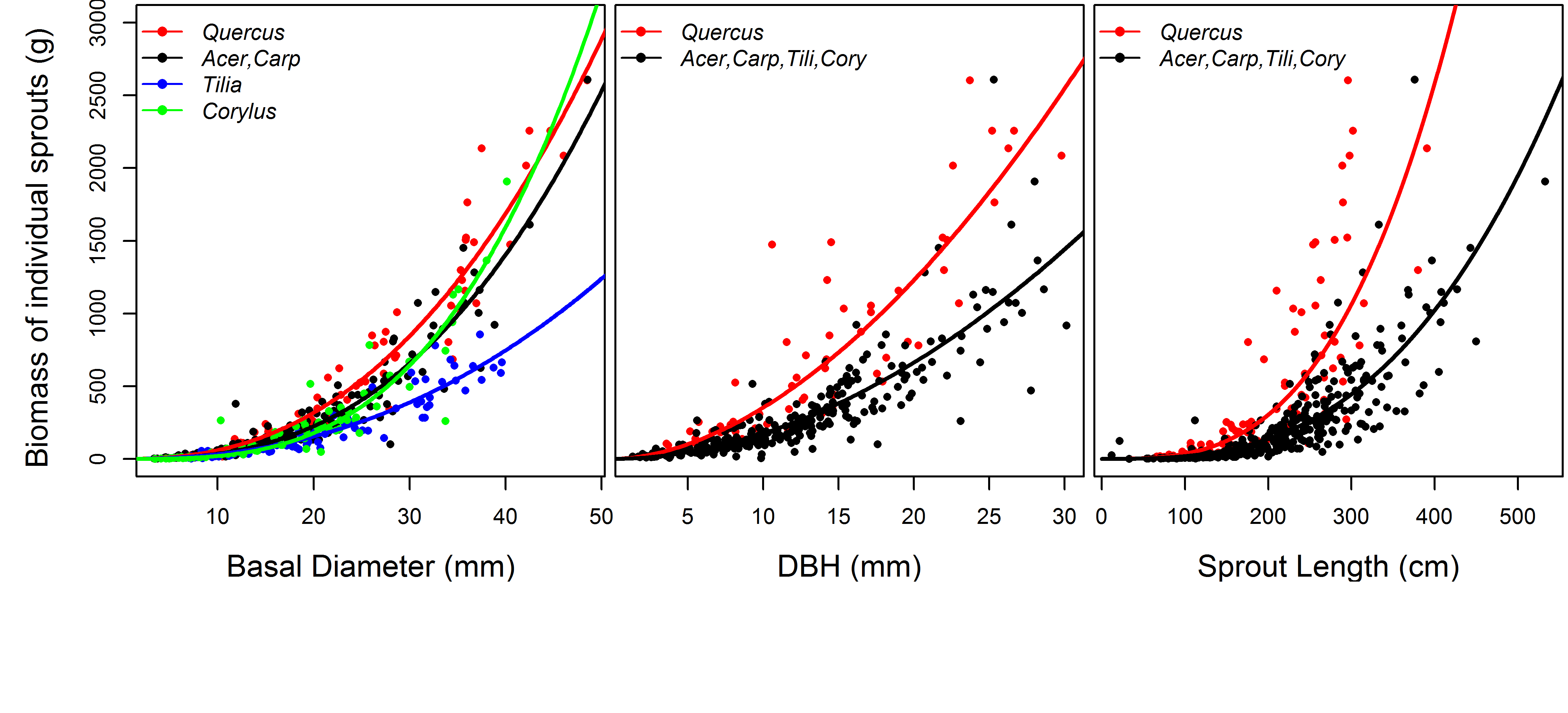

Supplement: S3 Fig — Power-law model was used for ploting lines. Species without significant difference in slope (P > 0.05) are shown together. (TIF) [file pone.0118388.s003.tif]
